# Public sphere attitudes towards the rumor sources of the COVID-19 pandemic: evidence from community perceptions in Iran

**DOI:** 10.1186/s12889-021-12254-x

**Published:** 2021-11-29

**Authors:** Morteza Banakar, Ahmad Kalateh Sadati, Leila Zarei, Saeed Shahabi, Seyed Taghi Heydari, Kamran Bagheri Lankarani

**Affiliations:** 1 Health Policy Research Center, Institute of Health, Shiraz University of Medical Sciences, Shiraz, Iran; 2 Department of Social Sciences, Yazd University, Yazd, Iran

**Keywords:** COVID-19, Rumor, Coronavirus, Misinformation, Crisis management

## Abstract

**Background:**

In the COVID-19 pandemic, rumors travel far faster than the outbreak itself. The current study aimed to evaluate the factors affecting the attitudes of individuals towards the rumors-producing media in Iran.

**Methods:**

An online cross-sectional survey was conducted in Iran in March 2020 on the source of information and rumors, along with the perception of individuals regarding the reasons for rumors propagation during the COVID-19 pandemic.

**Results:**

Results showed that the majority of the participants (59.3%) believed that social media were the main source of rumors. The lack of a reliable and formal news resource was also considered the most common cause of rumoring by the participants (63.6%). An evaluation was carried out to identify the main source of misinformation and rumors. Results showed that Retired participants considered foreign media (*P* < 0.001) as the main resource. The middle-income level participants believed that social media (*P* < 0.001) were the main source. In this regard, the highly educated participants (P < 0.001), government employees, and middle-income individuals (*P* = 0.008) believed that national media produced rumors.

**Conclusion:**

Although findings were achieved during the first peak of the COVID-19 pandemic, the authorities immediately introduced the national media as a reliable news resource, which allowed both media and its journalists to reduce the gap between themselves and the public sphere. It was suggested that social networks and foreign media be more accountable in pandemics.

**Supplementary Information:**

The online version contains supplementary material available at 10.1186/s12889-021-12254-x.

## Background

COVID-19 pandemic has become one of the major concerns of all nations globally, as it has affected many aspects of daily lives [1, 2]. A major part of mitigation strategies in this pandemic relies on community engagement. Solid information and credible social interaction are important in this regard, while factors including confusion, fear, panic, and misinformation or rumors have detrimental effects [2, 3]. There are no other choices than using non-pharmaceutical interferences to battle COVID-19, including social distancing and quarantine, risk communication, and information circulation; therefore, they have the highest importance in current pandemic management [4]. While media could be an important channel of communicating with society and increasing their engagement in the mitigation processes, it can also interfere with public health efforts if rumors are publicized [5]. The wrong or ambiguous information, which does not originate from reliable resources which do not deceive people, is called misinformation. It includes rumors, insults, and pranks [6]. Rumors consist of four distinct types. First of all, the legendary rumors are derived from stories or legends and contain popular fantasies in real-life events; they also characterize the supernatural beings, including various narratives from the SARS outbreak to traditional folklore stories and legends. Second, the aetiological narratives are baseless doubtful claims regarding the illness’s reasons, prevention, and therapies. Rumors of the second type could be perceived because of the nonexistence of enough information of the media regarding the properties of the novel virus at the first stages during the SARS period. The third type is known as protomemorates, which spread in a chain from one person to another. Nowadays, they are transmitted in the communities too much faster than in the past because of more powerful media. Finally, bogies are the last type of rumors that cause fear or anxiety in society. The rumors of city quarantine or food shortages are included in this category [7].

A study on the dimensions of SARS-related rumoring throughout China during an epidemic in 2003 showed a strong correlation between the scale of SARS infections and rumoring levels [7]. Another study in China during the COVID-19 pandemic revealed that the state media played an irresponsible role during the crisis [8]. Another study conducted by Cheung in West Africa during the EBOLA outbreak showed that rumors originated from the lack of information and fear [9]. Besides, community partnerships could prevent rumors, fear, and distrust, sometimes hiding family members’ illness or death [10].

There are both positive and negative impacts associated with social media. They could be implemented properly to change people’s behaviors and improve public health [11]. Moreover, social media can provide significant knowledge; therefore, it would be essential that people have appropriate access to social media during the COVID-19 pandemic and prevent rumoring [12]. Swamping the media with trustworthy data and information, purposeful media monitoring, and prompt response to rumors and misinformation are the most effective strategies to promote community engagement [13]. Although the recent technological advances have increased the data access of consumers by implementing various resources and networks, misinformation has begun to be spread worldwide, particularly through social media, during the pandemic due to the novelty of virus and avidity of communities for information (6). Accordingly, the virus concurrence and its viral news have led to faster rumoring than the outbreak itself [14, 15].

The Islamic Republic of Iran reported two COVID-19 deaths on February 18, 2020, 50 days after the first detected cases in China. Various social, economic, and political aspects could influence public health. Furthermore, the qualification of countries in COVID-19 management is influenced by political and economic states associated with positive and negative effects. Sanctions are the most influential political-economic factors with the highest limiting impacts on the capacity of Iran in pandemic management [16]. The Iranian Ministry of Health and Medical Education represents reports of the infected, recovered, and death cases every day; however, there are rumors represented by foreign media and cyberspace regarding the reported mortality rates, mass graves of dead cases, or considering the international airports of Iran as one of the potential centers of the outbreak. All of these factors have adverse effects on Iranian people’s general beliefs and attitudes towards pandemic management in the country [16, 17].

According to WHO, during the COVID-19 pandemic, we faced a new type of misinformation and rumor called infodemic. It includes excessive information, typically referring to a rapid and far-reaching spread of incorrect or misleading information on social media or mass media. Particularly, this misinformation led to the confusion of the public, legislators, and physicians. As Tedros Adhanom Ghebreyesus, WHO Director-General, said, “We’re not just fighting an epidemic; we’re fighting an infodemic.” However, no clear classification completely differentiates the rumors from each other [12, 18]. The information or misinformation achievement by the community was associated with considerable impacts on people’s behaviors during the pandemic. The current study aimed to evaluate the perception of individuals regarding COVID-19 rumors, detect the resources used by people to achieve data, and reveal the association between social factors and attitudes of individuals towards the source of rumors.

## Methods

### Study design and setting

Data was collected using an online cross-sectional study during 19-25 March 2020 in Tehran, Fars, Gilan, East Azarbaijan, Sistan and Baluchestan, and Isfahan Provinces of Iran. Table 1 provides more details of the surveyed provinces.

**Table 1:** Related information of surveyed provinces

Province	% of the total population of country ^a^	COVID-related information ^b^	
		Infection	Death
Tehran	16.6%	5098	512
Fars	6.07%	505	28
Gilan	3.17%	1191	461
East Azarbaijan	4.89%	813	159
Sistan and Baluchestan	3.47%	134	21
Isfahan	6.41%,	1979	336

^a^ Source: The last census of the Statistical Center of Iran (SCI);1395

^b^ Source: Center for Disease Control and Prevention, Ministry of Health and Medical Education (MOHME); Iran

The current investigation was conducted simultaneously with Nowruz, the thousands-year-old Persian new year celebration (March 21). Generally, people get prepared for this celebration from mid-February; therefore, streets become too crowded. Furthermore, most people prefer to travel during this 15-day holiday. It could be found from the mentioned facts that the risk of disease prevalence could peak due to the increase of interpersonal contacts during this period.

### Instruments and measures

In this study, a researcher-made questionnaire was applied to investigate the dominant media, information gathering, misinformation resources, the level of perceived misinformation, perception of individuals regarding the reasons of rumoring, and mechanisms of monitoring and controlling using the Likert scale. To measure the variables, relevant items were developed using both the literature and the experts’ opinions. The final questionnaire included seven items. Moreover, some questions gathered the participants’ demographics, including their age, gender, educational level, marital status, children, employment, socioeconomic status, and the effect of COVID-19 on their income. Supplementary file 1 is a blank copy of the mentioned questionnaire.

### Questionnaire validation

The first draft of the questionnaire was submitted to six academic experts in the research area. The questionnaire validity was evaluated during the meetings with these experts, including transparency, comprehensiveness, and items correlation. Therefore, some questions were modified considering their transparency and content. To ensure the measurement reliability, a pilot study was carried out in a setting of 60 participants before the commencement of the current study. Accordingly, a Cronbach’s alpha of 0.70 represented data reliability.

### Participants

At the beginning of the survey in each province, a focal point was selected as the starter to distribute the questionnaire link. The data was collected from Iranian people aged 18 years and over who had access to the internet. No sampling framework was used. The link was sent to anyone who could, whether answer anonymously or send it to others; hence, the questionnaire link was sent and rotated using the snowball method. In addition, an invitation letter and a written consent form, which included information about the research purposes and ethical issues, were provided for the individuals. In order to respect privacy and confidentiality, the questionnaires were designed anonymously, without receiving any identity information. A response validation rule was specified for each question to be answered according to the instructions, ensuring the lack of missing data; thus, the 2550 participants answered the questions properly.

### Statistical analysis

Data analysis was carried out through SPSS software version 18 (SPSS Inc., Chicago, IL, USA). Moreover, various factors were applied in order to describe data, including the mean, standard deviation, frequency, and percentage. The chi-square test was also applied to compare the sources of information and COVID-19 rumors regarding participants’ age, gender, education, employment, and socioeconomic status. Bonferroni adjustment takes 0.05/4 = 0.0125 of *P*-values as a corrected for the sources of information and COVID-19 rumors. The other significance level was set to P-values below 0.05.

## Results

The questionnaire was viewed 5000 times; however, only 2550 individuals completed the questionnaire. So, the completion rate in this study was nearly 50%. The mean age of participants was 36.38 ± 10.64 years. The study population consisted of 1246 men (48.9%) and 1304 women (51.1%). Moreover, 711 people (27.9%) were below 30 years of age, 1532 (60.1%) were between 30 to 50, and 307 (12%) were above 50 years of age.

According to the participants, social media, including WhatsApp, Telegram, Instagram, and the national broadcasting media, namely TV and radio, were the main sources of COVID-19 news. Furthermore, the newspaper was the least reported media (1.3% (32)) to achieve information. Social media was also considered as the primary source of misleading information for a majority of participants (59.3% (1513)); however, phone calls and text messages were regarded as the least rumor-containing media (4.5% (115)) (Table 2).

**Table 2:** The number (%) of each media usage with regard to COVID-19 information and misinformation

		National Media	Foreign Media	Social Media	Web	Newspaper	The phone call and text messages
It is your primary source of information regarding Covid-19:	No	1103 (43.4)	2139 (83.9)	1051 (41.2)	1887 (74.0)	2518 (98.7)	2403 (94.2)
	Yes	1447 (56.7)	411 (16.1)	1499 (58.8)	663 (26.0)	32 (1.3)	147 (5.8)
Most of the misinformation and rumors are related to this media:	No	1632 (64.0)	1496 (58.7)	1037 (40.7)	2167 (85.0)	2427 (95.2)	2435 (95.5)
	Yes	918 (36.0)	1054 (41.3)	1513 (59.3)	383 (15.0)	123 (4.8)	115 (4.5)
Total		2550 (100)	2550 (100)	2550 (100)	2550 (100)	2550 (100)	2550 (100)

Perceptions of participants regarding the main resources of rumors are presented in Table 3. According to findings, the lack of a reliable news resource was considered the most common cause of rumors (63.6% (1621)).

**Table 3:** The perceptions of participants regarding the main resources of rumors (Number (%))

		Lack of social media monitoring	Lack of reliable news source	Inaccuracy in choosing the news source	The uncertainty caused by the novelty of the disease	Other
It is the primary cause of the rumors:	No	1190 (46.7)	929 (36.4)	262 (49.5)	1348 (52.9)	2193 (86.0)
	Yes	1360 (53.3)	1621 (63.6)	1288 (50.5)	1202 (47.1)	357 (14.0)

Regarding the mechanism adopted to encounter rumors, most participants (24.1% (614)) mentioned that very few measures were adopted to tackle the rumors during the pandemic. Concerning COVID-19’s misleading information, most participants (31.3% (799)) mentioned that they became informed about the data uncertainty of this novel disease most of the time. Also, most of the participants (34.8% (888)) reported the moderate active monitoring and interventions of related organizations aimed at reducing the rumoring. Furthermore, most participants (33.1% (844)) reported that they often heard some news about the disease that was later disproved (Table 4).

**Table 4:** The perception of participants regarding COVID-19 rumors (n(%))

	Never	Rarely	Sometimes	Often	Always
How often there any mechanisms to take action against rumors?	452 (17.7)	614 (24.1)	902 (3.4)	386 (15.1)	196 (7.7)
How often had you been informed of the uncertainty of the information about Covid-19?	141 (5.5)	216 (8.5)	799 (31.3)	938 (36.8)	456 (17.9)
How often does an active organization monitor and respond to rumors?	446 (17.5)	584 (22.9)	888 (34.8)	429 (16.8)	203 (8.0)
Have you heard of any news about Covid-19, which has been later refuted?	126 (4.9)	320 (12.5)	639 (25.1)	844 (33.1)	621 (24.4)

Male participants reported using the websites as their main news resource more than females (*P* < 0.001). Moreover, participants over 50-year stated that they used the national and foreign media more frequently than other age groups (P < 0.001). Regarding the effect of the educational level on COVID-19 data resources, those with higher educational levels seemed to use foreign media, social media, and the web more frequently than the other groups (*P* = 0.003). The application of national media as the primary source of news was significantly more prevalent among individuals with a bachelor’s degree (*P* < 0.001). The type of employment also had a significant effect on the primary news resource for the participants. National media was significantly favored among retired people (72.5%) (*P* < 0.001), while freelancers (22.6%) reported foreign media as the favorite source of information (P < 0.001). Also, social media were more considered by the government employees (63.4%) (P < 0.001), and the web was the first common news resource for non-governmental employment (30.7%) (*P* = 0.007). Socioeconomic status did not significantly affect sources of information (*P* > 0.05) (Table 5).

**Table 5:** The number (%) of each media applied for COVID-19 news based on the demographic data of participants

		National Media			Foreign Media			Social Media			Web		
		No	Yes	*P*-value	No	Yes	*P*-value	No	Yes	*P*-value	No	Yes	*P*-value
**Sex**	M	550 (44.1)	696 (55.9)	0.377	1024 (82.8)	222 (17.8)	0.023	495 (39.7)	751 (60.3)	0.135	860 (69.0)	386 (31.0)	< 0.001*
	F	553 (42.4)	751 (57.6)		1115 (85.5)	189 (14.5)		556 (42.6)	748 (57.4)		1027 (78.8)	277 (21.2)	
**Age**	< 30	348 (48.9)	363 (51.1)	< 0.001*	618 (86.9)	93 (13.1)	< 0.001*	295 (41.5)	416 (58.8)	0.980	517 (72.7)	194 (27.3)	0.570
	30-50	653 (42.6)	879 (57.4)		1301 (84.9)	231 (15.1)		629 (41.1)	903 (58.9)		1145 (74.7)	387 (25.3)	
	> 50	102 (33.2)	205 (66.8)		220 (71.7)	87 (28.3)		127 (41.4)	180 (58.6)		225 (73.3)	82 (26.7)	
**Education**	Under diploma	43 (28.9)	106 (71.1)	< 0.001*	133 (89.3)	16 (10.7)	. < 0.001*	91 (61.1)	58 (38.9)	< 0.001*	122 (81.9)	27 (18.1)	0.003*
	Diploma	121 (38.9)	190 (61.1)		278 (89.4)	33 (10.6)		165 (53.1)	146 (46.9)		236 (75.9)	75 (24.1)	
	Associate’s degree	72 (36.9)	123 (63.1)		160 (82.1)	35 (17.9)		88 (45.1)	107 (54.9)		157 (80.5)	38 (19.5)	
	Bachelor’s degree	342 (40.2)	508 (59.8)		732 (86.1)	118 (13.9)		351 (41.3)	499 (58.7)		635 (74.7)	215 (25.3)	
	High educated	523 (50.2)	519 (49.8)		834 (80.0)	208 (20.0)		356 (34.2)	686 (65.8)		735 (70.5)	307 (29.5)	
**Employment**	Governmental employment	310 (40.8)	450 (59.2)	< 0.001*	651 (85.7)	109 (14.3)	< 0.001*	278 (36.6)	482 (63.4)	< 0.001*	571 (75.1)	189 (24.9)	0.007*
	Non-governmental employment	165 (45.2)	200 (54.8)		291 (79.7)	74 (20.3)		142 (38.9)	223 (61.1)		253 (69.3)	112 (30.7)	
	Freelancer	164 (51.4)	155 (48.6)		247 (77.4)	72 (22.6)		131 (41.1)	188 (58.9)		231 (72.4)	88 (27.6)	
	Student	187 (49.0)	195 (51.0)		339 (88.7)	43 (11.3)		148 (38.7)	234 (61.3)		268 (70.2)	114 (29.8)	
	Housewive	104 (33.9)	203 (66.1)		275 (89.6)	32 (10.4)		161 (52.4)	146 (47.6)		252 (82.1)	55 (17.9)	
	Retired	33 (27.5)	87 (72.5)		93 (77.5)	27 (22.5)		60 (50)	60 (50)		92 (76.7)	28 (23.3)	
	Unemployed	90 (51.4)	85 (48.6)		143 (81.7)	32 (18.3)		69 (39.4)	106 (60.6)		132 (75.4)	43 (24.6)	
	Daily-paid	41 (39.8)	62 (60.2)		84 (81.6)	19 (18..4)		53 (51.5)	50 (48.5)		75 (72.8)	28 (27.2)	
**Socioeconomic status**	High	270 (45.6)	322 (54.4)	0.134	492 (83.1)	100 (16.9)	0.512	246 (41.6)	346 (58.4)	.257	431 (72.8)	161 (27.2)	0.748
	Middle	466 (41.1)	667 (58.9)		944 (83.3)	189 (16.7)		447 (39.5)	686 (60.5)		841 (74.2)	292 (25.8)	
	Low	364 (44.6)	452 (55.4)		694 (85.0)	122 (15.0)		352 (43.1)	464 (56.9)		608 (74.5)	208 (25.5)	

*Base on Bonferroni adjustment P-value less than 0.0125 was significant

According to the findings, different groups mentioned various resources as the causes of rumoring. Male participants mostly considered social media (*P* = 0.008) as the source of rumors. Highly-educated individuals mostly reported national media as a source of rumors (*P* < 0.001). In other words, individuals with under diploma degrees (49% (73)) mostly reported foreign media as a source of rumors (P < 0.001). In contrast, highly-educated participants considered this media as a source of rumors less than others. For individuals aged 60 years and over, social media was considered a source of rumors (*P* = 0.005). On the other hand, individuals below 30 years of age considered the web as a source of rumors (*P* = 0.001). The freelancers (46.7% (149)) and the unemployed (45.7% (80)) participants mostly reported the national media as a rumoring resource (*P* < 0.001). A majority of the retired people (50.8% (61)) and housewives (45.0% (138)) mentioned the foreign media as a rumoring resource (*P* < 0.01). Individuals with middle income assumed social media as a rumoring resource (P < 0.001) (Table 6).

**Table 6:** The number (%) of each media as a source of rumors based on the demographic information of participants

		National Media			Foreign Media			Social Media			Web		
		No	Yes	*P*-value	No	Yes	*P*-value	No	Yes	*P*-value	No	Yes	*P*-value
**Sex**	M	783 (62.8)	463 (37.2)	0.233	700 (56.2)	546 (43.8)	0.013	474 (38.0)	772 (62.0)	0.008^a^	1042 (83.6)	204 (16.4)	0.062
	F	849 (65.1)	455 (34.9)		796 (61.0)	508 (39.0)		563 (43.2)	741 (56.8)		1125 (86.3)	179 (13.7)	
**Age**	< 30	451 (63.4)	260 (36.6)	0.554	429 (60.3)	282 (39.7)	0.419	328 (46.1)	383 (53.9)	0.001^a^	578 (81.3)	133 (18.7)	0.005^a^
	30-50	976 (63.7)	556 (36.3)		895 (58.4)	637 (41.6)		601 (39.2)	931 (60.8)		1322 (86.3)	210 (13.7)	
	> 50	205 (66.8)	102 (33.2)		172 (56.0)	135 (44.0)		108 (35.2)	199 (64.8)		267 (87.0)	40 (13.0)	
**Education**	Under Diploma	124 (83.2)	25 (16.8)	< 0.001^a^	76 (51.0)	73 (49.0)	< 0.001^a^	57 (38.3)	92 (61.7)	0.667	119 (79.9)	30 (20.1)	0.186
	Diploma	217 (69.8)	94 (30.2)		187 (60.1)	124 (39.9)		126 (40.5)	185 (59.5)		272 (87.5)	39 (12.5)	
	Associate’s degree	134 (68.7)	61 (31.3)		106 (54.4)	89 (45.6)		72 (36.9)	123 (63.1)		160 (82.1)	35 (17.9)	
	Bachelor’s degree	547 (64.4)	303 (35.6)		470 (55.3)	380 (44.7)		359 (42.2)	491 (57.8)		722 (84.9)	128 (15.1)	
	High educated	610 (58.5)	432 (41.5)		656 (63.0)	386 (37.0)		422 (40.5)	620 (59.5)		892 (85.6)	150 (14.4)	
**Employment**	Governmental employment	509 (67.0)	251 (33.0)	< 0.001^a^	420 (55.3)	340 (44.7)	0.009^a^	289 (38.0)	471 (62.0)	0.246	657 (86.4)	103 (13.6)	0.040
	Non-governmental employment	211 (57.8)	154 (42.2)		233 (63.8)	132 (36.2)		153 (41.9)	212 (58.1)		309 (84.7)	56 (15.3)	
	Freelancer	170 (53.3)	149 (46.7)		209 (65.5)	110 (34.5)		129 (40.4)	190 (59.6)		280 (87.8)	39 (12.2)	
	Student	249 (65.2)	133 (34.8)		229 (59.9)	153 (40.1)		155 (40.6)	227 (59.4)		301 (78.8)	81 (21.2)	
	Housewive	224 (73.0)	83 (27.0)		169 (55.0)	138 (45.0)		128 (41.7)	179 (58.3)		263 (85.7)	44 (14.3)	
	Retired	89 (74.2)	31 (25.8)		59 (49.2)	61 (50.8)		41 (34.2)	79 (65.8)		106 (88.3)	14 (11.7)	
	Unemployed	95 (54.2)	80 (45.7)		103 (58.6)	72 (44.1)		85 (48.5)	90 (51.5)		148 (84.5)	27 (15.4)	
	Daily-paid	71 (68.9)	32 (31.1)		63 (61.2)	40 (38.8)		47 (45.6)	56 (54.4)		87 (84.5)	16 (15.5)	
**Socioeconomic status**	High	340 (57.4)	252 (42.6)	0.001^a^	368 (62.2)	224 (37.8)	0.071	252 (42.6)	340 (57.4)	0.009^a^	505 (85.3)	87 (14.7)	0.729
	Middle	757 (66.8)	376 (33.2)		640 (56.5)	493 (43.5)		423 (37.3)	710 (62.7)		968 (85.4)	165 (14.6)	
	Low	528 (64.7)	288 (35.3)		483 (59.2)	333 (40.8)		357 (43.8)	459 (56.3)		687 (84.2)	129 (15.8)	

^a^Base on Bonferroni adjustment P-value less than 0.0125 was significant

## Discussion

The current study aimed to explain the attitudes of Iranian people towards rumors during the COVID-19 pandemic. Results revealed that social media, including WhatsApp, Telegram, Instagram, and national media such as IRI TV and radio, were the primary sources of COVID-19 news for the participants. In contrast to the findings of other investigations, Twitter did not have any role in Iran [13, 19].

Participants did not considerably use printed media (1.3% (32)) for COVID-19 news. This paradigm shift in the behaviors of consumers led to the innate features of these media platforms. In other words, the acquisition of information through social media platforms was more time-saving and cost-effective compared to conventional news media such as newspapers or television. Chatting and sharing the news with others through social media was found to be much easier [20]; also, it was the primary source of misleading information for most participants (59.3%(1513)).

In general, the inferential statistics regarding the relationship between social factors and attitudes towards the source of rumors, social networks, national media, and satellites were accused of forming rumors. In other words, the trust in news media and social media was dwindled [21]. Despite investigations in China during this SARS epidemic [8], Iran’s national media made efforts to represent clear news responsively. Community partnerships can prevent rumors, fear, and distrust [10], and this media should have a more closed relationship with people and the public sphere.

Socio-demographically, men were more likely than women to consider foreign media (*P* = 01) and social media (*P* = 00.8) as the rumoring resources. Regarding the rumors on the public health intervention, Kaler claimed that such skepticism would regularly lead to rumoring, influencing the thought processes or public health intervention. Theoretically, the widespread rumor of sterility could broadly articulate the shared understandings about reproductive bodies, collective survival, and global asymmetries of power [22]. This bio-power demonstrated the gender-based perceptions that were formed during the pandemic. The male participants of the current study were doubtful of foreign spaces, including the foreign media and social media, which led to the formation of concepts of overcoming the social discourse of the pandemic.

Considering the age, participants between 30 to 50 years of age assumed rumors were mainly resulted from social media (*P* = 0.001) and the web (*P* = 0.005), while > 50-year old participants were less concerned with social networks and the web. Individuals under 30 years of age also were not skeptical of cyberspace due to their higher existential connections with cyberspace. Moreover, individuals between 30 to 50 years of age use social media and the web more frequently. On the other hand, they held a skeptical view towards these spaces and considered social media and the web as rumor sources because of the generation gap. Individuals below 30 years of age had less generation gap; therefore, they did not feel alienated and held a positive attitude towards such spaces.

Increasing levels of education had a significant relationship with attitudes toward rumors in national (*P* < 0.001) and foreign (*P* = 0.002) media. Afassinou (2014) showed that improving the education level of the population could catalyze rumoring. In social networks, when people with higher educational levels heard a rumor in serious conflict with their beliefs, it was easier for them to counterattack the rumor and even do their best to prevent its propagation [23]. The current study showed that education could not affect participants’ attitudes towards the rumors from social networks and the web; also, it was found that educated individuals were in a more problematic position. It was believed that both media outlets were spreading the rumors. Due to the importance of education in such pandemics, the government must establish closer contacts with such individuals through the national media and spare its trust-building efforts.

Regarding employment, government employees believed that national media (*P* < 0.001) and foreign media (*P* = 0.009) produced rumors in pandemics, which was similar to the attitude held by educated individuals. Therefore, the authorities had to interact with their employees and attract their trust more actively in such situations than in the past. Considering the income status, the middle-income groups, whose income levels were equal to their expenses, believed that national media (*P* = 0.009) and social media (P = 0.009) produced rumors. It seemed that the critical view among the middle class was related to this perception. Further studies are recommended in this regard.

Rumors can significantly influence the control of pandemics [24]. Journalists have both built and undermined open belief, which is a valuable source of logical realities and a dangerous source of the rumor that intensifies the freeze [25]. Nowadays, modern media are a major source of news and data. One-third of the world’s population is engaged in social media, and others are entangled with the internet [26]. All of the mentioned media, such as social media, print media, and Twitter, can produce rumors [27].

On the other hand, social media consists of ubiquitous health misinformation, which is described as information that is not achieved from the greatest accessible evidence by medical experts [13]. Social media have become an effective and innovative channel of rumoring, influencing people’s lifestyles, thoughts, and values [28]. A qualitative study was carried out by Bastani et al. in Iran, which represented the lack of accurate monitoring of social media as the most important cause of rumoring. The current study revealed that the contribution of healthcare providers and authorities in improving public health literacy could control rumoring during the pandemic more efficiently [29]. Singh et al. showed that while there was a considerable enhancement in providing information about health issues, coronavirus, and the origin of the pandemic during the COVID-19 crisis, there were fewer arguments about rumors and myths. However, misinformation and rumors play a pivotal role in pandemics [13]. One of the main reasons for COVID-19 rumoring is that most people share information on social networks regardless of its accuracy. Pennycook et al. showed that people could distinguish true and false news if they consider the correctness of information [30]. The authorities should identify and amplify the help-seeking information, donations, and notifications required for the public; they also have to detect and counter the blames or rumors to improve the crisis information publishing strategies in the future [31].

The current study showed the necessity of building more social trust by authorities during the pandemics. It should be noted that based on the experiences achieved during the COVID-19 pandemic in Iran, a serious dilemma was formed between social network satellite and national media. In the first phase of the pandemic, foreign media worked hard to provide the news and analyses of the pandemic’s origins in Iran. It was due to the coincidence of the outbreak, national celebrations, and elections in Iran. The news was soon republished on social media. The primary purpose was to express the political weakness and incompetence of the government, which led to the skepticism of the public and serious doubts regarding the national media. However, the politicians could solve this problem partially through solidarity and focusing on the national media. From the outset, the national media was referred to by the government as a source of COVID-19 news. A spokesman for the Ministry of Health announced the latest new cases, recovered cases, and mortality of COVID-19 at News 14:00 daily. Therefore, the national media gradually became the main source of COVID-19 statistics. However, many journalists tried harder to verify foreign and social media rumors and consequently clarify the information.

### Study limitations

The main limitation of this study is that the current study was conducted at the beginning of the outbreak when duality was formed between national media on the one hand and foreign media and social networks on the other. The second limitation is that due to the cross-sectional design of this study, only correlations were investigated. In addition, the current study may not be a representative sample of all population groups, particularly the individual that have no access to the internet and the illiterate individual. So, there is a possibility of selection bias. Therefore, different data collection strategies should be implemented to ensure that all population’ groups are included and the data collected are representative. Finally, due to this pandemic’s rapid and sudden occurrence, another limitation of this study was the lack of validated measures. There is a risk of measurement bias, and the objectivity of the measured concept may be questionable. Therefore, the other measurement strategies could be used for the assessment to be valid and reliable.

## Conclusion

Since rumors have adverse effects on citizens’ mental health and crisis management, news management during the outbreak is one of the most critical social issues that policymakers should consider. Failure to tackle rumors could lead to the ineffectiveness of pandemic policies. Many of the measures of news management have been performed via national media despite powerful competitors such as foreign and social media. Providing accurate news for all ages and gender groups with different educational backgrounds can help policymakers overcome the rumors. What seems to be of paramount importance is to build trust between the government and the public in the pandemics. This issue is suggested to be examined in future studies. As the main governance tool in large-scale pandemics, the national media requires more trust and closeness to the public sphere.

## Supplementary Information

**Additional file 1.**

## Data Availability

The datasets used and/or analyzed during the current study available from the corresponding author on reasonable request.

## Abbreviations

WHO: World health organization

## References

[1] Munster VJ, Koopmans M, van Doremalen N, van Riel D, de Wit E (2020). A novel coronavirus emerging in China—key questions for impact assessment. N Engl J Med.

[2] Organization WH. Responding to community spread of COVID-19: interim guidance, 7 march 2020: World Health Organization; 2020.

[3] Organization WH (2020). Risk communication and community engagement ( RCCE) readiness and response to the 2019 novel coronaviruses ( 2019- nCoV): interim guidance, 26 January 2020.

[4] Wilder-Smith A, Freedman D (2020). Isolation, quarantine, social distancing and community containment: pivotal role for old-style public health measures in the novel coronavirus (2019-nCoV) outbreak. J Travel Med.

[5] Depoux A, Martin S, Karafillakis E, Preet R, Wilder-Smith A, Larson H. The pandemic of social media panic travels faster than the COVID-19 outbreak. Journal of Travel Medicine. J Travel Med. 2020;27(3):taaa031. 10.1093/jtm/taaa031 PMC7107516 32125413

[6] Lazer DM, Baum MA, Benkler Y, Berinsky AJ, Greenhill KM, Menczer F, Metzger MJ, Nyhan B, Pennycook G, Rothschild D (2018). The science of fake news. Science.

[7] Tai Z, Sun T (2011). The rumouring of SARS during the 2003 epidemic in China. Sociol Health Illness.

[8] Jinqiu Z (2003). The SARS epidemic under China’s media policy. Media Asia.

[9] Cheung EY (2015). An outbreak of fear, rumours and stigma: psychosocial support for the Ebola virus disease outbreak in West Africa. Intervention.

[10] Marais F, Minkler M, Gibson N, Mwau B, Mehtar S, Ogunsola F, Banya SS, Corburn J (2015). A community-engaged infection prevention and control approach to Ebola. Health Promot Int.

[11] Sahni H, Sharma H (2020). Role of social media during the COVID-19 pandemic: beneficial, destructive, or reconstructive?. Int J Acad Med.

[12] Ali S, Khalid A, Zahid E. Is COVID-19 immune to misinformation? A brief overview. Asian Bioeth Rev. 2021;13(2):1-23. 10.1007/s41649-020-00155-x PMC7985752 33777228

[13] Singh L, Bansal S, Bode L, Budak C, Chi G, Kawintiranon K, et al. A first look at COVID-19 information and misinformation sharing on Twitter. ArXiv [Preprint]. 2020:arXiv:2003.13907v1.

[14] Larson HJ (2018). The biggest pandemic risk?. Viral Misinform Nat.

[15] McCauley M, Minsky S, Viswanath K (2013). The H1N1 pandemic: media frames, stigmatization and coping. BMC Public Health.

[16] Rassouli M, Ashrafizadeh H, Shirinabadi Farahani A, Akbari ME. COVID-19 Management in Iran as One of the Most Affected Countries in the World: Advantages and Weaknesses. Front Public Health. 2020;8:510. 10.3389/fpubh.2020.00510 PMC7533538 33072688

[17] Tuite AR, Bogoch II, Sherbo R, Watts A, Fisman D, Khan K (2020). Estimation of coronavirus disease 2019 (COVID-19) burden and potential for international dissemination of infection from Iran. Ann Intern Med.

[18] Zarocostas J (2020). How to fight an infodemic. Lancet.

[19] Shahi GK, Dirkson A, Majchrzak TA (2021). An exploratory study of COVID-19 misinformation on twitter. Online Soc Netw Media.

[20] Shu K, Sliva A, Wang S, Tang J, Liu H (2017). Fake news detection on social media: a data mining perspective. ACM SIGKDD Explorations Newsletter.

[21] Dubois E, Minaeian S, Paquet-Labelle A, Beaudry S (2020). Who to Trust on Social Media: How Opinion Leaders and Seekers Avoid Disinformation and Echo Chambers. Soc Media Soc.

[22] Kaler A (2009). Health interventions and the persistence of rumour: the circulation of sterility stories in African public health campaigns. Soc Sci Med.

[23] Afassinou K (2014). Analysis of the impact of education rate on the rumor spreading mechanism. Physica A: Statistical Mechanics and Its Applications.

[24] Okware S, Omaswa F, Zaramba S, Opio A, Lutwama J, Kamugisha J, Rwaguma E, Kagwa P, Lamunu M (2002). An outbreak of Ebola in Uganda. Tropical Med Int Health.

[25] Thomas K (2020). What should health science journalists do in epidemic responses?. AMA J Ethics.

[26] Ortiz-Ospina E. The rise of social media. https://ourworldindata.org/rise-of-social-media. Accessed 23 Nov 2021.

[27] Dang A, Smit M, Moh'd A, Minghim R, Milios E: Toward understanding how users respond to rumours in social media. In Proc. IEEE/ACM Int. Conf. Adv. Social Netw. Anal. Mining (ASONAM). 2016: 777-84.

[28] Zhu L, Zhao H, Wang H (2019). Stability and spatial patterns of an epidemic-like rumor propagation model with diffusions. Phys Scr.

[29] Bastani P, Bahrami MA. COVID-19 Related Misinformation on Social Media: A Qualitative Study from Iran. J Med Internet Res. 2020. 10.2196/18932. Epub ahead of print. 10.2196/18932 32250961

[30] Pennycook G, McPhetres J, Zhang Y, Lu JG, Rand DG (2020). Fighting COVID-19 misinformation on social media: experimental evidence for a scalable accuracy-nudge intervention. Psychol Sci.

[31] Li L, Zhang Q, Wang X, Zhang J, Wang T, Gao T-L, Duan W (2020). Tsoi KK-f, Wang F-Y: characterizing the propagation of situational information in social media during COVID-19 epidemic: a case study on weibo. IEEE Transact Comput SocSyst.

