# Supplementary material for: Public sphere attitudes towards the rumor sources of the COVID-19 pandemic: evidence from community perceptions in Iran
Source: BMC Public Health. 2021 Nov 29;21:2187. doi: 10.1186/s12889-021-12254-x (PMC8628274; doi:10.1186/s12889-021-12254-x)
Supplement: Supplementary file 1 — Additional file 1. [file 12889_2021_12254_MOESM1_ESM.docx]

**The esteemed participant,**

The present questionnaire aims to contribute to the existing knowledge on the factors affecting citizens' attitudes toward Iran's rumor-producing media during the COVID-19 outbreak. It takes approximately 10 minutes to complete this questionnaire. Your participation in this study is entirely voluntary. The information and data are strictly confidential. We will publish only aggregated, anonymous results.

Thank you for your support!

**Answer Guide:**

Please, choose the most suitable option in your opinion.

Table Q.1: Demographic profile.

| **Age (years):** |
| --- |
| **Gender:** |
| **Marital status:** |
| **Education:** |
| **Employment:** |
| **Location:** |
| **Expenditure Ratio:**  Expenditure> Income ☐  Expenditure= Income ☐  Expenditure< Income ☐ |
| **Tel/ Phone (Optional):** |

Table Q.2: The questionnaire.

| **How do you mainly follow Corona/COVID-19 news?** (You can choose more than one option)   - National TV and/or Radio - Foreign media/ satellite - Social networks (WhatsApp, Telegram, Facebook, etc.) - Internet/Web - Newspapers and other publications - Telephone/ SMS |
| --- |
| **In your opinion, which media has the most rumors and false information?** (You can choose more than one option)   - National TV and/or Radio - Foreign media/ satellite - Social networks (WhatsApp, Telegram, Facebook, etc.) - Internet/Web - Newspapers and other publications - Telephone/ SMS |
| **How often warned about the uncertainty of information about COVID-19 because it is unknown?**   - Always - Often - Sometimes - Rarely - Never Top of Form |
| **How often heard the news about COVID-19 that has subsequently been denied?**   - Always - Often - Sometimes - Rarely - Never |
| **What do you think might be causing the rumors?** (You can choose more than one option)   - Top of Form - Bottom of Form - Lack of monitoring of social networks. - Lack of reliable news source. - Inaccuracy in checking the source of the news. - Uncertainties arising from the novelty of the disease - Other (please explain) |
| **Are there active monitoring mechanisms and/or responsive organizations** **to rumors?**   - Top of Form - Bottom of Form - Always - Often - Sometimes - Rarely - Never |
| **Are there mechanisms to deal with rumors?**   - Always - Often - Sometimes - Rarely - Never |
